# Multi-modality imaging evaluation of recurrent Tako-tsubo syndrome in a patient with coronary artery fibromuscular dysplasia

**DOI:** 10.1186/s12947-017-0117-4

**Published:** 2017-11-30

**Authors:** Yun Cheng, Chenying Lu, Kan Liu

**Affiliations:** State University of New York, Upstate Medical University, Syracuse, NY 13202 USA

## Abstract

**Background:**

Integrated bedside and sophisticated cardiac imaging techniques help characterize the discrepancy between myocardial injury and mechanic dysfunction in acute myocardial infarction.

**Case presentation:**

A 57 year-old woman presented with sudden onset chest pain and ventricular fibrillation after hearing of her brother’s death. The electrocardiography indicated “anterior wall ST segment elevation myocardial infarction”. Coronary angiography ruled out obstructive lesion in the major coronary arteries, but revealed fibromuscular dysplasia of the distal left anterior descending artery. The ventriculography showed remarkable ventricular dilation, which affected much broader myocardium than the culprit vessel supplied. In a subsequent cardiac magnetic resonance study, delayed contrast (gadolinium) image revealed a focal left ventricular (LV) apical infarction. Her LV systolic function normalized within 1 week, except for a residual apical hypokinesis. She developed recurrent chest pain and LV dilation when she was laid off 9 months later. After supportive therapy, her symptoms improved and LV dysfunction normalized again.

**Conclusions:**

“Tako-tsubo” syndrome can occur recurrently in the heart with pre-existing localized myocardial infarction. Its molecular mechanism and clinical significance warrants further investigation.

## Background

Although its clinical manifestation often mimics that of an acute anterior wall myocardial infarction (MI), Takotsubo syndrome (TTS) was postulated to have completely different etiology, pathophysiology and prognosis [1, 2]. As more real-time cardiac imaging, particularly bedside echocardiography, are applied, TTS have been frequently identified in patients with co-existing coronary artery disease. Multi-modality imaging evaluation help characterize the discrepancy between myocardial injury and ventricular mechanic dysfunction during TTS and MI.

## Case presentation

**Fig. 1:**
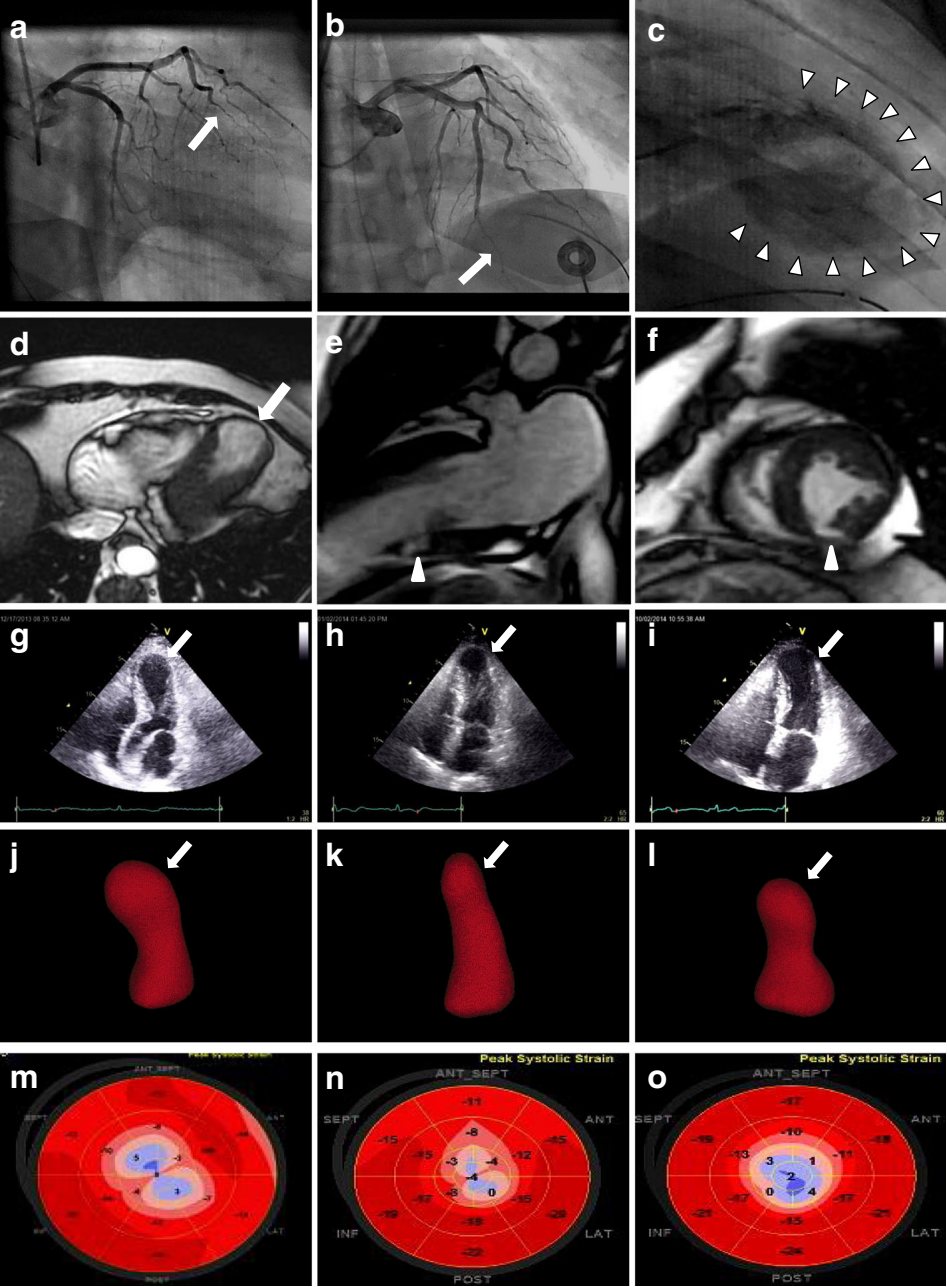
Recurrent Tako-tsubo syndrome (TTS) in a woman with coronary artery fibromuscular dysplasia and left ventricular apical infarction. **a** (right anterior oblique caudal projection) and (**b**) (right anterior oblique cranial projection): The coronary angiograms showing a tapering and smooth narrowing (with a discrete transition from normal to diseased artery) in distal left anterior descending artery (arrows) .**c** The Ventriculography (right anterior oblique projection) demonstrating apical ballooning involving broader myocardium beyond the territory of distal LAD (arrowheads). **d** Non-contrast cardiac magnetic resonance (CMR) images indicating dilated left ventricle (arrow). **e** Two chamber view contrast CMR with late gadolinium enhancement (LGE) image revealing localized inferoapical myocardial infarction (arrowhead). **f** Short-axis view contrast CMR with LGE image revealing localized inferoapical apical myocardial infarction (arrowhead). **g**-**o** Recurrent TTS with alternated ventricular contractile patterns. **g**, **h**, **i** Two dimensional echocardiograms; (**j**, **k**, **l**): Three-dimensional echocardiograms; M,N,O: Speckle tracking echocardiograms (automated function imaging with a “bull’s eye” plot). **g**, **j**, and **m**: Initial TTS; (**h**), (**k**) and (**n**): recovery period; (**i**), (**l**), and (**o**): recurrent TTS

A 57 year-old woman presented with sudden onset chest pain/ventricular fibrillation after hearing of her brother’s death. The electrocardiography indicated “anterior wall ST segment elevation myocardial infarction”. Laboratory data was notable for cTnT 1.12 ng/mL. Coronary angiography ruled out obstructive lesion in the major coronary arteries, but revealed a tapering and long narrowing distal left anterior descending artery (LAD, Fig. 1a and b), which was consistent with angiographic feature of coronary artery fibromuscular dysplasia [3]. The ventriculography showed remarkable ventricular dilation, which affected much broader myocardium than the culprit vessel supplied (Fig. 1c, arrowheads). In a subsequent cardiac magnetic resonance (CMR) study, left ventricular (LV) remained dilated (Fig. 1d). Delayed contrast (gadolinium) image confirmed a localized mayocardial infarction in the inferoapical wall (Fig. 1e and f).

One week later, repeated transthoracic echocardiography (TTE) showed nearly normalized LV systolic function except for a residual apical hypokinesis (Fig. 1h–n) compared with original episode (Fig. 1g–m). Nine months later, when she was laid off, she developed recurrent chest pain and significant LV dilation, with different contractile pattern (Fig. 1i–o). With only supportive therapy, both her symptoms and LV dysfunction spontaneously improved quickly.

## Discussion

The present case illustrates a unique scenario, in which TTS occur recurrently in the heart with pre-existing localized myocardial infarction. In contrast to “gate-keeper” role of bedside echocardiography, advanced cardiac imaging has improved the tomographic visualization and our understanding on myocardial injury/metabolism in both TTS [4, 5] and MI [6]. During acute MI, psychological stress and/or physical pain can stimulate central/autonomic nervous systems, and increase bioavailability of cortisol and circulating catecholamines, which may affect the myocardium supported by both culprit and non-culprit coronary arteries.

In the presence of coronary artery disorder, is hypocontractile myocardium beyond the myocardial territory of the culprit coronary artery a coincidence, or a cause-and-effect? Also, its clinical significance remains unknown. Molecular research has revealed that TTS is mediated by a cardio-protective switching of epinephrine signaling transduction through the pleiotropic β_2_-adrenergic receptors [7]. Whether the TTS during and/or after MI represents a universal physiologically adaptive response of a jeopardized heart warrants further investigation.

## Conclusions

TTS occurs recurrently in a patient with pre-existing localized myocardial infarction caused by coronary artery fibromyscular dysplasia.

## Abbreviations

CMR: Cardiac magnetic resonance
ECG: Electrocardiogram
LAD: Left anterior descending artery
MI: Myocardial infarction
STEMI: ST segment myocardial infarction
TTE: Transthoracic echocardiography
TTS: Tako-tsubo syndrome
